# Using Social Media and Technology to Communicate in Pediatric HIV Research: Qualitative Study With Young Adults Living With or Exposed to Perinatal HIV

**DOI:** 10.2196/20712

**Published:** 2020-06-23

**Authors:** Claire A Berman, Deborah Kacanek, Mindy Nichamin, Dominique Wilson, Mariam Davtyan, Liz Salomon, Kunjal Patel, Megan Reznick, Katherine Tassiopoulos, Sonia Lee, Jose Bauermeister, Mary Paul, Theresa Aldape, George R Seage III

**Affiliations:** 1 Department of Epidemiology Harvard TH Chan School of Public Health Boston, MA United States; 2 Center for Biostatistics in AIDS Research Harvard TH Chan School of Public Health Boston, MA United States; 3 Department of Pediatrics University of Southern California Los Angeles, CA United States; 4 Westat Rockville, MD United States; 5 Maternal and Pediatric Infectious Disease Branch Eunice Kennedy Shriver National Institute of Child Health and Human Development Bethesda, MD United States; 6 Center for AIDS Research University of Pennsylvania School of Nursing Philadelphia, PA United States; 7 Texas Children’s Hospital Baylor College of Medicine Houston, TX United States

**Keywords:** pediatric HIV, perinatal HIV, youth, young adults, social media, study retention, COVID-19

## Abstract

**Background:**

As young adults living with perinatal HIV (PHIV) or perinatal HIV exposure but uninfected (PHEU) grow older and manage the challenges and competing demands of young adulthood, new approaches are needed to facilitate their retention in longitudinal research and clinical care beyond in-person clinic visits. Severe acute respiratory syndrome coronavirus 2 (SARS-CoV-2), the novel virus that causes coronavirus disease (COVID-19), emerged in the United States in January 2020 and has underscored this need; studies are adapting to remote communication with and data collection from participants. However, there are limited data on communication preferences among young adults who are living with PHIV or PHEU.

**Objective:**

The objectives of this qualitative study were to describe participants’ perceptions and use of social media and technology in their personal lives and in the context of participating in longitudinal pediatric HIV research and to describe the implications of the use of technology and social media for communication and retention purposes within a longitudinal pediatric study about HIV.

**Methods:**

We conducted 6 focus group discussions with 31 young adults living with PHIV and 13 in-depth interviews with 6 young adults living with PHIV and 7 living with PHEU. We asked about their preferences for the use of social media and digital technology in the Adolescent Master Protocol, a US-based longitudinal cohort study of youth affected by HIV.

**Results:**

Participants’ willingness to use social media platforms, telephone calls, SMS text messages, and video calls within the context of HIV research varied due to fears of HIV stigma and inadvertent disclosure. However, trusting relationships with clinical staff positively impacted their willingness to use these platforms.

**Conclusions:**

Our findings offer insight into how pediatric studies and clinics can communicate with participants as they age, even as new technologies and social media platforms emerge and replace old ones. For optimal retention, pediatric clinical staff should consider communication approaches offering flexible and tailored options for young adults participating in HIV research.

## Introduction

As young adults living with perinatal HIV (PHIV) or perinatal HIV exposure but uninfected (PHEU) grow older and manage the challenges and competing demands of young adulthood, new approaches are needed to facilitate their retention in longitudinal research studies and clinical care beyond in-person clinic visits. Recent events have underscored this need. Severe acute respiratory syndrome coronavirus 2 (SARS-CoV-2), the novel virus that causes coronavirus disease (COVID-19), emerged in the United States in January 2020 and has quickly forced studies to adapt their procedures to enable remote communication with and collect data from participants. Young adults living with PHIV or PHEU may be navigating increased autonomy, careers, school, and relationships while managing HIV, stigma, and disclosure [1-4], medication fatigue from lifelong antiretroviral regimens [5], and family relationships in the context of HIV. Using digital communication methods holds potential for retaining the participation of these young adults in studies. Although their aging has coincided with a rapid expansion of health care and health communication technology options, little is known about their specific communication preferences in the context of HIV research.

The evidence demonstrating the effectiveness of strategies utilizing social media or digital technology to retain youth living with HIV in research is limited [6]. However, an increasing number of health promotion and peer-to-peer programs have engaged youth living with HIV using text messaging, websites, games, and smartphone apps [7-14]. The general success of these methods may indicate some willingness among youth living with HIV to engage with social and digital media around HIV prevention, treatment-related communication, and peer support. However, these interventions have mostly focused on youth who acquired HIV later in life, who may differ in key health outcomes from youth who acquired HIV perinatally [15-17]. It is not well understood whether young adults living with PHIV or PHEU have unique communication preferences due to lifelong experiences with HIV, antiretrovirals, and stigma, and this topic is in need of further exploration.

The Adolescent Master Protocol (AMP) of the Pediatric HIV/AIDS Cohort Study (PHACS) network [18] follows the physical, cognitive, social, and behavioral development of youth and adolescents living with PHIV and PHEU in the US. Due to declining retention rates in AMP as participants approach young adulthood, there is urgent need to optimize communication with young adults in PHACS for their continued study participation. PHACS therefore launched the AMP Up protocol for young adults in early 2014; this protocol uses a flexible internet-based and clinic-based approach [19]. Novel communication approaches that respond to participants’ preferences are critical to ensure that PHACS can continue its research into the long-term impact of PHIV and antiretroviral exposure among young adults living with PHIV or PHEU.

To our knowledge, no other study has examined specific digital communication and information preferences from the direct perspectives of youth living with PHIV and PHEU. The objectives of this qualitative study were to describe participants’ perceptions and use of social media and technology in their personal lives and in the context of participating in longitudinal pediatric HIV research and to describe the implications of the use of technology and social media for communication and retention purposes within a longitudinal pediatric study about HIV. It is our hope that the insights described below will help inform digital strategies to retain participants and patients in study protocols and care during and beyond the COVID-19 pandemic.

## Methods

This formative qualitative study was conducted among young adults living with PHIV or PHEU at PHACS AMP clinics in 8 cities across the United States: New York, New York; Denver, Colorado; Philadelphia, Pennsylvania; Fort Lauderdale, Florida; San Diego, California; Houston, Texas; Chicago, Illinois; and San Juan, Puerto Rico.

### Recruitment and Sampling

Young adults were eligible for this study if they were between 18 and 25 years of age; living with PHIV and aware of their HIV status or living with PHEU and aware of their biological mother’s HIV status; enrolled at the time in AMP or receiving HIV care at a clinic with experience participating in an HIV-related longitudinal study in the past four years; and able to provide written informed consent in either English or Spanish. PHACS clinic staff at local AMP sites recruited participants using purposive sampling [20].

Institutional Review Boards at each site and the Harvard TH Chan School of Public Health approved the protocol for this qualitative study. All participants provided written informed consent in their preferred language (English or Spanish).

### Data Collection

From June 2013 to May 2014, trained facilitators (CB, DK, and JB) conducted 6 focus group discussions with 31 young adults living with PHIV and one-on-one in-depth interviews with 6 young adults living with PHIV and 7 living with PHEU. The focus groups ranged in size from 3 to 8 (mean 5) participants. Six young adults living with PHIV opted for a one-on-one interview instead of participating in a focus group. All young adults living with PHEU participated in one-on-one interviews due to insufficient numbers of eligible youth per site to hold focus groups. All participants completed a 90-minute audio-recorded focus group discussion or interview followed by a 15-minute self-administered survey. The survey assessed demographics, access to digital technology, social media preferences, and frequency of use.

One or two of the three facilitators trained in qualitative research named above conducted the focus group discussions and interviews. In Puerto Rico, the interviews were conducted by a Spanish-speaking facilitator (JB). At each clinic, the focus group discussions and interviews were held in private rooms. Facilitators used a structured discussion guide covering the following topics: personal use of and access to mobile phones, computers, the internet, and social media; perceptions of adulthood; attitudes towards connecting with other young adults affected by HIV; participant concerns and hopes in their current stage of life; information needs and preferences during their transition to adulthood; motivations for participating in PHACS and HIV research; willingness to use social media to communicate with PHACS; and design and content preferences for a new private website portal for study participants.

### Data Analysis

Focus groups and interviews were audio recorded and transcribed verbatim in English, except for those held in Puerto Rico, which were transcribed verbatim in Spanish and then translated into English. To preserve anonymity, all identifying data were removed. Therefore, the quotations in this paper only identify participants’ HIV status and whether the response was recorded during a focus group or one-on-one interview. Facilitators took notes and prepared debriefing reports to record insights into dynamics that were not easily captured from the transcripts alone.

Analysis of the survey data included calculating percentages and frequencies of the sociodemographic characteristics of the participants and their digital communication and device use and preferences. Analysis of the qualitative data used a thematic approach [21,22]. All transcripts were coded by the study team using Atlas.ti software [23]. Drawing themes from both focus groups and interviews, the team developed a codebook, which was refined iteratively. The first 20% of the transcripts were double-coded to assess and ensure consistency across coders; discrepancies in coding were resolved by updating codebook definitions or creating new codes where necessary, and the final codes were entered into Atlas.ti and applied to all transcripts.

## Results

### Characteristics of the Participants

Of the 44 young adults living with PHIV or PHEU participating in the focus groups and one-on-one interviews, 40 (91%) completed the study survey. Of these 40 participants, 26 (65%) identified as female, 35 (87%) were aged 18 to 21 years, 25 (63%) identified as Black/African American, 15 (38%) identified as Hispanic/Latino, 33 (83%) were living with PHIV, and 7 (17%) were living with PHEU (Table 1).

**Table 1:** Selected participant characteristics (N=40).

Characteristic		n (%)
**Gender**		
	Male	14 (35)
	Female	26 (65)
	Transgender	0 (0)
**Age (years)**		
	18 to 21	35 (87)
	22 to 24	5 (13)
**Race**		
	White	9 (23)
	Black/African American	25 (63)
	American Indian	2 (5)
	Native Hawaiian/Pacific Islander	2 (5)
	Other	6 (15)
Hispanic/Latino		15 (38)
**HIV status**		
	PHIV^a^	33 (83)
	PHEU^b^	7 (17)

^a^PHIV: perinatal HIV.

^b^PHEU: perinatal HIV exposure but uninfected.

### Personal Use of Social Media and Technology

Young adults reported frequent use of smartphones and computers to access the internet (Table 2).

A few participants reported limited internet access due to poor network connectivity, lack of home Wi-Fi connectivity, or lack of an internet-enabled device. Most reported accessing the internet “everywhere” on devices they carried, and they “never” or “rarely” used a work or public library computer. Most reported using Facebook and Instagram; some cited Tumblr, Skype, or Twitter, as well as more solitary forms of entertainment (eg, YouTube and video games). One focus group participant with PHIV described the factors that influence which platform they used:

If it’s a quick message, somebody might text someone—send a Facebook message…whereas with, like if you’re having an event or something, you can post it on Facebook…or put it on Twitter.

**Table 2:** Frequency of device use among young adults (N=40).

Device type	Frequency, n (%)					
	Daily	Weekly	Monthly	A few times	Any use^a^	Never
Mobile phone	28 (70)	0 (0)	2 (5)	0 (0)	8 (20)	2 (5)
Home computer	11 (28)	4 (10)	3 (8)	7 (18)	3 (8)	12 (30)
School computer	0 (0)	4 (10)	2 (5)	7 (18)	0 (0)	27 (68)
Work computer	1 (3)	1 (3)	2 (5)	0 (0)	0 (0)	36 (90)
Friend or family member’s computer	2 (5)	5 (13)	2 (5)	8 (20)	1 (3)	22 (55)
Public computer	0 (0)	2 (5)	1 (3)	9 (23)	1 (3)	27 (68)
Other device	1 (3)	0 (0)	0 (0)	0 (0)	0 (0)	39 (98)

^a^Any use: use is indicated but the frequency is not specified.

Attitudes towards different forms of social media varied. For instance, while some participants mentioned using Twitter as a way to keep up to date with news (eg, activities of celebrities they liked), most expressed negative attitudes towards it. Of all social media platforms, Facebook elicited the most divided opinions. While some participants stated that they liked Facebook, others said that Facebook was no longer popular among people their age and that they used Facebook somewhat reluctantly to keep in touch with people in their lives. Conversely, Instagram was identified as more popular and was viewed by many participants as a positive way to connect with people, the optimal way to promote a brand, more tailored to user preferences, more “personal” than Facebook, and more entertaining. Most participants stated they did not use email as often as texting. Some perceived email as a more “serious” form of communication compared to texting and reserved it for work, while others had created separate email addresses for personal use, work, and school. The participants described using phone apps if they were easy to navigate; however, they noted that apps often ran too slowly, froze, or were unintuitive to navigate. Regarding this point, one focus group participant with PHIV also noted:

It just depends on what kind of website you’re looking at…or what you actually want to do on that website. ’Cause, like, sometimes, like, the mobile version’s more hassle than if you just went to the desktop version and do it the long way.

### Phone and Device Sharing Among Peers

Many young adults reported phone-sharing (passing their mobile phones back and forth while spending time together) among peers. Because of this, many expressed concern that their HIV-affected status could be inadvertently disclosed if their peers saw smartphone alerts from texts, emails, or social media posts about HIV, including study-related messages. One focus group participant with PHIV stated:

Say you’re at your friend’s house, or whatever…and the PHACS [study] thing pops up on your phone, and then your friend’s sitting next to you, they’ll know about you. And that’s, like, how the disclosure part come up in there.

Young adults living with PHIV expressed stronger fear than those living with PHEU of inadvertent disclosure of their HIV-affected status, although some young adults living with PHEU also mentioned concerns that their mother’s HIV status, or their own HIV-affected status, could be disclosed.

### Privacy and Confidentiality Concerns

Many participants expressed concern about using digital forms of communication in the context of a long-term study about HIV due to privacy and confidentiality concerns. Some expressed feeling less inclined to visit a social media page on their mobile phones in public due to concerns that their online activity could be viewed by others. Many were averse to seeking information about HIV on their mobile phones in public for the same reason. Young adults reported managing their online presence carefully, aware that privacy protections could quickly change as the Terms of Service on social media sites were updated. For this reason, most participants were hesitant or unwilling to join even a private and invisible Facebook group related to HIV. For some, this concern outweighed the potential benefits of using social media to connect with peers living with HIV. Regarding this point, one interview participant with PHIV stated:

My concern is confidentiality there. Never know who’s going to…many things can happen. You can lose your phone, people go through your phone…I’ve had my email hacked before…That shit happens.

### Relationship With Clinic Staff

Participants reported higher willingness to use texting or social media to stay in touch with study staff with whom they had close and trusting relationships. Having participated in research throughout their lives, many young adults described strong connections to PHACS clinical staff. These young adults described staff as “family” they looked forward to seeing during study visits. One focus group participant with PHIV stated:

They know everything about you, know everything you’ve ever done…known you since birth…it literally is your second family.

Another interview participant with PHEU noted:

…’cause these people here are like my family. Like, they’ve known me from the minute I came out of the womb.

Another focus group participant with PHIV stated:

…I have a doctor that I can—that’s been taking care of me since I was a baby…and to see the doctor smile that you’ve known since you were a baby…it feels good, because you know you’re still alive, and they see you’re still alive and you’re still here surviving and living.

The high level of trust in these longstanding relationships was cited by participants as a significant factor in their willingness to use social media and texting to communicate in an HIV-related study.

### Communication Preferences Within Research

Most participants stated that they preferred that study staff contact them by telephone or email (Table 3), which they perceived as more private and secure.

**Table 3:** Communication preferences of young adults in PHACS in personal life vs in pediatric HIV research (N=40).

Communication type		Frequency, n (%)				
		Very often	Somewhat often	Not often	Any use^a^	Never
**Texting**						
	Use in personal life^b^	26 (65)	4 (10)	0 (0)	5 (13)	5 (13)
	Preferred use with the study^c^	9 (23)	9 (23)	1 (3)	1 (3)	20 (51)
**Email**						
	Use in personal life	2 (5)	9 (23)	9 (23)	1 (3)	19 (48)
	Preferred use with the study	11 (28)	9 (23)	1 (3)	5 (13)	14 (36)
**Facebook**						
	Use in personal life	20 (50)	9 (23)	0 (0)	3 (8)	8 (20)
	Preferred use with the study	5 (13)	3 (8)	3 (8)	0 (0)	29 (73)
**Twitter**						
	Use in personal life	2 (5)	2 (5%)	6 (15)	1 (3)	29 (73)
	Preferred use with the study	1 (3)	0 (0%)	3 (8)	0 (0)	36 (90)
**Telephone call**						
	Use in personal life	18 (45)	6 (15)	2 (5)	3 (8)	11 (28)
	Preferred use with the study	7 (18)	4 (10)	4 (10)	5 (13)	20 (51)

^a^Any use: use is indicated but the frequency is not specified.

^b^Survey question: “How often do you and your friends use the following to communicate with each other?”

^c^Survey question: “How often would you prefer to get study-related information from PHACS using the following?”

Some participants expressed a willingness to text or message privately on a social media platform, as they trusted clinic staff to ensure the messages would be secure. However, many stated that they would not feel comfortable using social media to stay in contact with an HIV-related study due to fear of inadvertent disclosure of their HIV-affected status. Some participants expressed that omitting the acronym “HIV” and using code words for the study (or framing messages as simple “appointment reminders”) was a compromise that might increase their comfort with written modes of communication. Others stated that this would still feel intrusive or risky, possibly inviting uncomfortable questions from their peers.

Some participants reported reluctance to communicate about the study outside of the clinic because they preferred to forget about HIV in their everyday lives. As one focus group participant with PHIV noted:

See, when I’m not [at the clinic], I forget that I have HIV at all…So I never, like, have a need to look it up. I don’t know if that makes sense. But I’m never, like, curious about something…’cause I’m like, “Nah.” It’s not in, like, the front of my mind at all, ever.

As another interview participant with PHIV stated:

The only thing I don’t like about the study is that it reminds me…it’s a blast to the past of where this all started with me….80% is me helping, the other 20% is me being reminded [of how] this all started.

When asked whether they would take advantage of a new flexible study format that would allow them to take surveys online from home and reduce clinic visits, the participants’ responses were mixed. Some participants stated they would still want to come to the clinic to complete their surveys, while others felt they would benefit from the remote option. Participants who preferred taking surveys remotely expressed that it was convenient, time-saving, and cost-effective. However, those who preferred to complete surveys in the clinic expressed a desire to visit with study staff and ensure the privacy of their responses.

Participants expressed a desire to access study information, especially information on findings from the research they participated in (eg, lay summaries of study results, study announcements). Regarding this point, one interview participant with PHEU stated:

I just like feeling like I’m a part of something, like…bigger than me.

As another focus group participant with PHIV stated:

I would like to know…because we do these [studies]…and I would like to know how that information benefitted you, because you never hear about what happened afterwards…and how it helped.

Participants also stated a desire for a private app or website for the study participants that would not be solely focused on HIV. Most expressed a desire for resources related to young adulthood, including employment, school, housing, health care and health insurance, and maintaining healthy relationships.

## Discussion

### Principal Findings

While previous studies have more broadly illuminated the experiences of youth living with HIV, our study investigated the unique preferences of young adults living with PHIV or PHEU regarding social media use in longitudinal pediatric HIV research communication. Our findings suggest that tailoring both the mode and content of communication to participants’ individual preferences, as well as remaining flexible to allow changes in communication preferences over time, may yield better study retention. In this qualitative study, young adults living with PHIV or PHEU reported frequent use of internet-enabled devices (especially smartphones) and social media in their personal lives, mirroring young adults in the United States more generally [24,25]. However, they expressed varying degrees of willingness to engage with these technologies in the context of an HIV-focused study. For some, a private Facebook message felt acceptable; for others, SMS text messages or email were preferable; others were comfortable only with a telephone call to minimize the digital trail of their connection to HIV. In our study, a fear of inadvertent disclosure of an HIV-affected status through social media or technology use was consistent with other studies of youth living with HIV [11,26]. Most participants expressed caution about connecting with an HIV study online. Participants living with PHIV expressed strong concerns about HIV stigma and inadvertent disclosure, similar to other youth living with HIV worldwide [1-4,27-30], more often than participants living with PHEU. These concerns, combined with the common practice of sharing devices and actively engaging with each other’s social media accounts, influenced their social media and technology preferences in the context of longitudinal HIV research.

As with previous research into transition from pediatric to adult health care among young people living with HIV [31-36], participants in this study reported close relationships with their pediatric providers and study staff. Some expressed that their established relationships with clinicians mitigated privacy concerns and increased their willingness to consider using social media and texting to communicate with trusted providers about HIV research. However, many also expressed preferences for private communication channels. Studies should therefore provide staff with the time and resources they need to build and maintain trusting relationships with participants. Study staff should also consider offering participants flexible communication options, asking for consent first, using coded language in messages to protect privacy, tailoring messages to specific participants, and giving participants the option to change their communication preferences over time. It is critical for staff to be aware that even seemingly benign texts or social media messages from study staff that do not mention HIV may be perceived as intrusive or distressing by young adults living with PHIV or PHEU. These messages may still trigger questions from peers, with whom they may share smartphones and other devices. Importantly, using these methods of communication in the context of a pediatric HIV study carries the double risk of disclosing not only the young adult participant’s but also their mother’s HIV status.

Young adults living with PHIV explicitly named various aspects of emerging young adulthood beyond their health and HIV as key priorities in life, such as housing, employment, school, and relationships, building on previous research and resources focused on living and coping with an HIV diagnosis [5,7-9,31-36]. Many participants expressed a desire to receive results from the studies they participate in. Study staff should consider discussing or offering resources and information on these topics as part of remote communication.

The results of our study underscore the myriad ways that individuals may experience being affected by HIV from birth. Having grown up with a mother living with HIV and having potentially been receiving antiretroviral treatment since childhood, some young adults living with PHIV or PHEU may wish to avoid thinking about HIV in their day-to-day lives. Others may find HIV to be a strong focus in their lives that feels either neutral or even positive. Young adults’ responses emphasize the importance of mirroring their varied experiences in communication strategies. Communication that focuses holistically on individuals within a broader constellation of joys, transitions, and challenges inherent to emerging young adulthood—and which provides young adults with the autonomy to decide how HIV fits into this constellation—may be most effective when considering approaches to study retention.

### Limitations

There are a number of limitations of this research. The final transcripts of the focus group discussions denoted only male and female-sounding voices, limiting the ability to contextualize key themes according to race, age, gender identity, or location. While our sample was demographically similar to the overall population of the AMP Up protocol [37], participants who were willing to participate in focus groups or interviews may not be representative of the general PHACS population or of participants in other pediatric HIV studies in their views and opinions. For example, it is possible that they have experienced and navigated stigma differently or have had different opportunities to develop coping strategies compared to study participants who chose not to join our study. We conducted this research at only 8 of the 14 PHACS AMP sites; participants in unrepresented parts of the country may have different access to the internet, prefer different communication methods, or operate within a different local context of HIV stigma. The majority of PHACS sites are located in urban settings, and of all eligible youth, those living closer to the clinic may have been more likely to participate. The results of this qualitative study, conducted in 2013 and 2014, may not reflect current attitudes or newer technologies and social media platforms; however, the results can inform fundamental steps staff can take to safely introduce the use of newer and evolving technologies as study communication tools.

### Conclusions

Our findings offer insights into how HIV-focused studies can communicate with young adult participants even as new technologies and social media platforms emerge and replace old ones. While participants in our study were young adults living with PHIV or PHEU engaged in longitudinal pediatric HIV research, our results could inform strategies for using social media and technology to recruit new participants into HIV research or treatment. They could also be applied to retaining young adults living with PHIV or PHEU in care during and after the COVID-19 pandemic, should remote communication and telehealth become an enduring norm in HIV care. Finally, these results may have implications for health communication approaches with young adults managing other perinatally or nonhorizontally acquired chronic illnesses or culturally stigmatized diagnoses.

## Abbreviations

AMP: Adolescent Master Protocol
COVID-19: coronavirus disease
PHACS: Pediatric HIV/AIDS Cohort Study
PHIV: perinatal HIV
PHEU: perinatal HIV exposure but uninfected
SARS-CoV-2: severe acute respiratory syndrome coronavirus 2
